# Effect of magnesium sulfate administration on blood–brain barrier in a rat model of intraperitoneal sepsis: a randomized controlled experimental study

**DOI:** 10.1186/cc3004

**Published:** 2004-11-23

**Authors:** Figen Esen, Tulin Erdem, Damla Aktan, Mukadder Orhan, Mehmet Kaya, Haluk Eraksoy, Nahit Cakar, Lutfi Telci

**Affiliations:** 1 Professor, University of Istanbul, Istanbul Faculty of Medicine, Department of Anesthesiology and Intensive Care, Istanbul, Turkey; 2 Staff Anesthesiologist, University of Istanbul, Istanbul Faculty of Medicine Department of Anesthesiology and Intensive Care, Istanbul, Turkey; 3 MD, University of Istanbul, Istanbul Faculty of Medicine Department of Anesthesiology and Intensive Care, Istanbul, Turkey; 4 Professor, University of Istanbul, Istanbul Faculty of Medicine Department of Physiology, Istanbul, Turkey; 5 Professor, University of Istanbul, Istanbul Faculty of Medicine, Department of Infectious Disease and Clinical Microbiology, Istanbul, Turkey

**Keywords:** blood–brain barrier, brain edema, magnesium, sepsis, septic encephalopathy

## Abstract

**Introduction:**

Permeability changes in the blood–brain barrier (BBB) and their possible contribution to brain edema formation have a crucial role in the pathophysiology of septic encephalopathy. Magnesium sulfate has been shown to have a protective effect on BBB integrity in multiple experimental models. In this study we determine whether magnesium sulfate administration could have any protective effects on BBB derangement in a rat model of sepsis.

**Methods:**

This randomized controlled experimental study was performed on adult male Sprague–Dawley rats. Intraperitoneal sepsis was induced by using the infected fibrin–thrombin clot model. To examine the effect of magnesium in septic and sham-operated rats, a dose of 750 μmol/kg magnesium sulfate was given intramuscularly immediately after surgery. Control groups for both infected and sham-operated rats were injected with equal volume of saline. Those rats surviving for 24 hours were anesthetized and decapitated for the investigation of brain tissue specific gravity and BBB integrity by the spectrophotometric assay of Evans blue dye extravasations. Another set of experiments was performed for hemodynamic measurements and plasma magnesium level analysis. Rats were allocated into four parallel groups undergoing identical procedures.

**Results:**

Sepsis significantly increased BBB permeability to Evans blue. The dye content of each hemisphere was significantly lower in the magnesium-treated septic rats (left hemisphere, 0.00218 ± 0.0005; right hemisphere, 0.00199 ± 0.0007 [all results are means ± standard deviation]) than in control septic animals (left hemisphere, 0.00466 ± 0.0002; right hemisphere, 0.00641 ± 0.0003). In septic animals treated with magnesium sulfate, specific gravity was higher (left hemisphere, 1.0438 ± 0.0007; right hemisphere, 1.0439 ± 0.0004) than in the untreated septic animals (left hemisphere, 1.0429 ± 0.0009; right hemisphere, 1.0424 ± 0.0012), indicating less edema formation with the administration of magnesium. A significant decrease in plasma magnesium levels was observed 24 hours after the induction of sepsis. The dose of magnesium that we used maintained the baseline plasma magnesium levels in magnesium-treated septic rats.

**Conclusions:**

Magnesium administration attenuated the increased BBB permeability defect and caused a reduction in brain edema formation in our rat model of intraperitoneal sepsis.

## Introduction

Patients with severe sepsis often manifest symptoms of encephalopathy. Acute alterations in mental status, which occur fairly frequently in septic patients, have been shown to be associated with poor prognosis [1]. However, not much is known about the exact mechanism of brain injury in sepsis. Studies have suggested that septic encephalopathy might involve a disturbance of plasma and brain neutral amino acid transport across the blood–brain barrier (BBB), similar to those seen in porto-systemic encephalopathy. This process has been related to the breakdown of the BBB because patients with septic encephalopathy have high protein levels in the cerebrospinal fluid [2]. Recently, derangements in the BBB causing perivascular edema have been demonstrated in sepsis-induced pigs [3].

Protective effects of magnesium sulfate (MgSO_4_) against BBB breakdown after severe insulin-induced hypoglycemia have been reported in animals [4]. Similar effects of magnesium on BBB were also evident in a diffuse traumatic brain injury model in rats [5-7].

In summary, MgSO_4 _was shown to have a protective effect on BBB integrity in multiple experimental models. We hypothesized that MgSO_4 _will also protect against BBB derangements observed in sepsis and tested the hypothesis in a rat model of sepsis induced by an intraperitoneally inserted infected fibrin–thrombin clot.

## Methods

One hundred and twenty-six male Sprague–Dawley rats weighing 320–440 g were used in this study. Rats were purchased from the Institute for Experimental Research and Application (Istanbul Medical Faculty), and were cared for before and during all stages of the experimental protocol in compliance with the applicable institutional guidelines and regulations of the Institute for Experimental Medicine Research and Application.

Rats were prepared for surgery under anesthesia with intramuscular 100 μg/g ketamine (Parke-Davis, Morris Plains, NJ, USA) and 20 μg/g xylazine hydrochloride Rompun 2% (Bayer, Munich, Germany) and allowed to breathe spontaneously. The loss of corneal reflex and no movement in response to a painful stimulus confirmed maintenance of adequate anesthesia for the experimental procedure. The rats were subsequently randomized into one of four groups: sham control (C), sham control MgSO_4_-treated (C-Mg), septic (S) and septic with MgSO_4 _(S-Mg).

Intraperitoneal sepsis was induced with the infected fibrin–thrombin clot model described by Mathiak and colleagues [8]. Fibrin–thrombin clots were formed by adding 2 ml of 1% sterile fibrinogen solution, 1 ml of a bacterial suspension (1.8 × 10^9 ^colony-forming units/ml [infected] or vehicle [sterile 0.9% NaCl]) and 160 μl (100 units/ml) of sterile human thrombin to a 5 ml syringe. The resulting clot was then incubated at room temperature for 30 min before implantation into the abdominal cavity. The *Escherichia coli *strain was isolated from an intra-abdominal collection from a patient with secondary peritonitis. The bacteria were inoculated into a brain heart infusion broth (DIFCO Laboratories, Detroit, MI, USA) and incubated overnight at 35°C. The count of *E. coli *was adjusted to 1.8 × 10^9 ^colony-forming units/ml with McFarland standard 6. After making a 0.5 cm midline abdominal incision, the peritoneum was opened and the prepared clot was injected into the peritoneal cavity directly from the syringe. Sham-operated rats had a sterile clot injected into their peritoneal cavity. To examine the effect of magnesium in septic and sham-operated rats, a dose of 750 μmol/kg MgSO_4 _was given intramuscularly immediately after surgery. Control groups for both infected and sham-operated rats were injected with an equal volume of saline.

After surgery, the animals were given 50 μl/g per hour of saline subcutaneously and were allowed to wake up while breathing spontaneously. They were returned to their cages and were allowed free access to water. Those rats surviving for 24 hours after the surgery were anesthetized and decapitated for the investigation of brain tissue specific gravity (SG) and BBB integrity.

We used the method described by Mikawa and colleagues [9] to determine BBB integrity by Evans blue (EB) dye. EB dye (4 ml/kg, 2%) was administered intravenously and allowed to circulate for 60 min. The animals were then perfused with saline through the left ventricle at a pressure of 110 mmHg until colorless fluid was obtained from the right atrium. Afterwards, the brains were removed and dissected. Each hemisphere was weighed and the samples were then homogenized in 3.5 ml phosphate-buffered saline and vortex-mixed for 2 min after the addition of 2.5 ml of 60% trichloroacetic acid to precipitate protein. The samples were then cooled for 30 min and centrifuged for 30 min at 1000 r.p.m. The absorbance of the supernatants for EB dye was measured at 610 nm with a spectrophotometer. EB dye content is expressed as μg/mg of brain tissue against a standard curve.

The method defined by Marmarou and colleagues was used for the determination of SG [10]. We obtained 1 mm^3 ^samples taken from the right and left hemispheres of each animal. Samples were placed into linear density gradient columns of kerosene and bromobenzene. A calibration curve was determined for each column by using anhydrous K_2_SO_4 _solutions of known SG (1.045, 1.040, 1.035 and 1.025). Brain tissue SG values were subsequently determined with this calibration curve.

Another set of experiments were performed for hemodynamic measurements and plasma magnesium level analysis. These rats were allocated into four parallel experimental groups with identical procedures. Right femoral artery catheterization was performed under general anesthesia for blood pressure monitoring and blood sampling. Blood samples (0.5 ml) were taken for the determination of plasma magnesium levels at baseline (T0) and 24 hours (T24) after the induction of sepsis, and an equal volume of saline was given. Mean arterial pressure was recorded at baseline and 2, 3, 4, 8, 12 and 24 hours after the surgical procedure. Four of 12 rats in group S and 3 of 11 rats in group S-mg died within 24 hours of the induction of sepsis. Data for these rats were excluded from the study. We continued to enter rats with a balanced randomization sequence until we had eight surviving rats for each group.

### Statistical analysis

The results are expressed as means ± standard deviation. EB dye content, brain tissue SG, serum magnesium levels, mean arterial pressures and heart rates were compared among four groups with a Kruskal–Wallis analysis of variance followed by Dunn's multiple comparisons test. A Mann–Whitney *U*-test and a Friedman nonparametric repeated-measures test were used for within-group comparisons. Paired serum magnesium levels were compared within each group by using a Wilcoxon signed rank test. Mortality rate was compared between septic groups receiving and not receiving magnesium with a χ^2 ^test. A probability (*P*) of less than 0.05 was considered significant.

## Results

Thirteen of 29 rats in group S and 10 of 26 rats in group S-Mg died within 24 hours after the induction of sepsis, whereas all of the rats in groups C and C-Mg survived. The mortality rate was not statistically different between septic rats receiving and not receiving magnesium (in the experimental groups, χ^2 ^= 0.229, *P *= 0.632; in the monitoring groups, χ^2 ^= 0.100, *P *= 0.752). Both groups of septic rats appeared ill as demonstrated by exudates around nose and eyes, tachypnea and decreased spontaneous movement. Sham-operated rats seemed grossly normal and were active within their cages.

**Figure 1:**
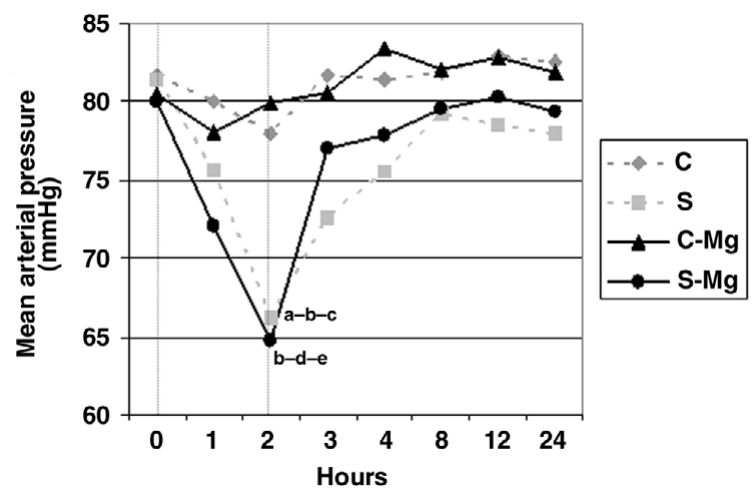
Hemodynamic data. Groups: sham control (C, *n *= 8), sham control MgSO_4_-treated (C-Mg, *n *= 8), septic (S, *n *= 8) and septic MgSO_4_-treated (S-Mg, *n *= 8). Mean arterial pressures compared among four groups using a Kruskal–Wallis analysis of variance followed by Dunn's multiple comparisons test. ^a^Septic versus sham control, *P *< 0.05. ^b^Septic versus sham control MgSO_4_-treated, *P *< 0.05. ^c^At 2 hours after the induction of sepsis versus baseline value (in the septic group), *P *< 0.01. ^d^Septic MgSO_4_-treated versus sham control MgSO_4_-treated, *P *< 0.01. ^e^At 2 hours after the induction of sepsis versus baseline value (in the septic MgSO_4_-treated group), *P *< 0.05. A Friedman nonparametric repeated-measures test was used for within-group comparisons.

Changes in mean arterial pressure are summarized in Figure 1. A significant decrease was observed 2 hours after the induction of sepsis in groups S and S-Mg. No further changes in blood pressures were observed with the administration of magnesium in the control and sepsis groups.

Plasma magnesium levels were comparable between groups at baseline (Table 1). A significant decrease in plasma magnesium levels was observed 24 hours after the induction of sepsis. An intramuscular dose of 750 μmol/kg MgSO_4 _maintained the baseline plasma magnesium levels in magnesium-treated septic rats.

Quantitative estimation of the EB dye revealed that sepsis significantly increased BBB permeability as measured by EB extravasations into brain tissue. In the S-Mg group, BBB permeability was significantly decreased in comparison with the S group (Table 2).

The SG of both hemispheres taken from sepsis-induced rats were significantly less than the sham-operated rats, indicating the formation of brain edema after the induction of sepsis (Table 3). Brain tissue SG measurements in the magnesium-treated septic rats were significantly higher than in the untreated sepsis group. Within-group comparisons indicated no difference between the right and left hemispheres.

## Discussion

The results of the present study demonstrate that treatment with magnesium immediately after experimental sepsis attenuated BBB permeability and the extent of brain edema formation.

Alterations of BBB permeability with subsequent brain edema formation are common features of septic encephalopathy. Several hypotheses for the pathogenesis of septic encephalopathy have been discussed in the literature: metabolic derangement, direct bacterial invasion of the central nervous system, the effect of endotoxin on the brain, or altered cerebral macrocirculation and microcirculation [11-16]. Recent evidence implicates the changes in the BBB permeability that favor brain edema formation in the pathophysiology of septic encephalopathy [3,17]. In our model the BBB permeability defect induced by sepsis, as demonstrated by the EB dye extravasation technique, is consistent with previous reports demonstrating a loss of BBB integrity as a result of a septic challenge; however, the change in the SG representing brain tissue edema formation was relatively minor. Although the small change in SG that we obtained in the sepsis group reached statistical significance, indicating some amount of edema formation with the induction of sepsis, it is not possible to relate the edema formation to the disturbed integrity of the BBB.

Our results are consistent with previous reports on the integrity of the BBB and the role of a permeability defect in the formation of cerebral edema using other models of cerebral damage [18-20]. In our previous experimental study we evaluated the effects of magnesium on brain edema formation and BBB breakdown after closed-head trauma in rats [5]. Our results of BBB breakdown by the measurement of EB dye extravasation were comparable with those that we obtained in our sepsis model; however, the changes in SG were higher in the traumatic brain injury model than in our sepsis model. This might be explained by the different mechanisms causing BBB breakdown and edema formation in trauma and sepsis. The discrepancy between brain edema and BBB permeability defect in sepsis might also indicate a low grade of permeability defect due to the complex cascade of sepsis, which is not enough to create edema as such in trauma. Another possible explanation might be that the quantitative determination of BBB permeability defect by EB dye extravasation is more sensitive than the SG method for determining brain edema.

Other methods have been used to determine BBB damage in septic encephalopathy. In rodents with sepsis, colloidal iron dioxide [21], ^14^C-labelled amino acids [22] and ^125^I-labelled albumin [23] have been shown to pass from the circulation into the brain parenchyma in a similar manner to that seen in portosystemic encephalopathy. However, there is no evidence in the literature to suggest that this damage is related to edema formation in sepsis. Most recently, morphologic changes have been showed in the frontal cortex of a pig model of sepsis [3]. Fecal peritonitis resulted in severe perimicrovessel edema that was associated with swelling and rupture of astrocyte endfeet. Although this was suggested as evidence for the breakdown of the BBB, the ultrastructure of intercellular tight junctions seemed morphologically intact in pigs with sepsis. The authors have suggested that some other mechanism might be involved in the formation of edema. It is not known whether edema formation is related to BBB breakdown or other factors in sepsis. The exact mechanism and the relation between BBB breakdown and edema formation in sepsis-induced brain injury need to be further evaluated by more sensitive methods.

A major finding of the present study is that magnesium administration attenuates the increase in BBB permeability and edema formation. The exact mechanism of magnesium's beneficial effect on the integrity of the BBB is unclear. However, magnesium can affect many aspects of the mediator cascade that can cause a permeability defect in the BBB. Alternatively, magnesium can act directly on the BBB. Magnesium's cytoprotective effect to reduce the profound breakdown of the BBB was first demonstrated in a rat model of severe insulin-induced hypoglycemia [4]. In this study it was speculated that magnesium might exert this effect through suppression of the endothelial cells. It was suggested that even before magnesium reaches the brain site it interacts with the endothelial cells forming the BBB and inhibits their activation [4,24,25]. To our knowledge, the present data are the first to show the positive effects of magnesium on sepsis-induced BBB permeability changes. Although this might have clinical significance, a contrary suggestion could be that increasing the integrity of the BBB might also have negative effects in terms of antibiotic emergence when the clinical situation is complicated with encephalitis or meningitis. In our model of intra-abdominal sepsis, the cultures of brain specimens taken after the experiment were all sterile (data not shown).

One of the major pitfalls in the interpretation of the data was the difficulty of establishing a dose response for magnesium. In our present study the dose and the timing of magnesium administration were chosen with reference to our previous experiments on traumatic brain injury [5]. This dose of magnesium was determined as an optimum dose showing the best neurologic outcome in a traumatic brain injury model [26]. Plasma magnesium levels decreased significantly with the induction of sepsis and returned to nearly control levels with the dose of magnesium that we administered. However, it is known that the plasma magnesium level does not represent tissue magnesium content, and the lack of correlation between plasma magnesium and total body magnesium content in healthy subjects has already been reported [27]. More recently, it was demonstrated that free magnesium levels in brain tissue is a sensitive method that reflects magnesium homeostasis in a traumatic brain injury model [28]. Although we do not know to what extent the plasma magnesium levels represent brain tissue levels in the present study, our data show that significant beneficial effects are achievable with the dose administered. However, future studies will be needed to establish a dose response by measuring free magnesium levels in brain tissue for the effects of magnesium therapy in sepsis-induced brain injury.

## Conclusion

This investigation shows that sepsis increases BBB permeability and leads to the formation of brain edema in septic rats. Magnesium administration attenuated the increased BBB permeability and caused a reduction in brain edema formation in our rat model of intraperitoneal sepsis. The precise mechanisms and the pharmacodynamics of magnesium administration in sepsis-induced brain injury need further investigation.

## Key messages

• 	Sepsis causes BBB permeability defect.

• 	Magnesium attenuates the increased BBB permeability associated with sepsis.

## Abbreviations

BBB = blood–brain barrier; EB = Evans blue; SG = specific gravity.

## Competing interests

The author(s) declare that they have no competing interests.

## Authors' contributions

All authors were responsible for study design and implementation of the experiment. Study data were collected by TE, DA and FE. Results were analyzed by FE and TE. The manuscript was written by FE and TE; all authors participated in revisions and gave approval to the final draft for submission for publication.

## Figures and Tables

**Table 1:** Plasma magnesium concentrations

Measurement	Group (*n*)	T0	T24	*P*
Plasma Mg (mM)	C (8)	1.11 ± 0.05	1.10 ± 0.05	NS
	S (8)	1.09 ± 0.05	0.89 ± 0.06^a,b^	0.0078
	C-Mg (8)	1.10 ± 0.06	1.29 ± 0.06	0.0078
	S-Mg (8)	1.13 ± 0.03	1.01 ± 0.08^c^	0.0156
KW		2.708	26.863	
d.f.		3	3	
*P*		>0.05	<0.0001	

Abbreviations: d.f., degrees of freedom; KW, Kruskal–Wallis test statistic; NS, not significant; *P*, approximate χ^2 ^*P *value; T0, basal measurement; T24, measurement at 24 hours. Groups: sham control (C), sham control MgSO_4_-treated (C-Mg), septic (S) and septic MgSO_4_-treated (S-Mg). Data are expressed as means ± standard deviation.

Dunn's multiple comparisons test: ^a^septic versus sham control, *P *< 0.05; ^b^septic versus sham control MgSO_4_-treated, *P *< 0.001; ^c^septic MgSO_4_-treated versus sham control MgSO_4_-treated, *P *< 0.01. Paired serum magnesium levels were compared within groups using a Wilcoxon signed rank test. Two-tailed *P *values are shown in the last column.

**Table 2:** Assessment of blood–brain barrier permeability by Evans blue dye content in brain tissue

Measurement	Group (*n*)	Left hemisphere	Right hemisphere	P
EB dye (μg/g)	C (8)	0.00160 ± 0.0003	0.00145 ± 0.0003	0.33
	S (8)	0.00466 ± 0.0002^a,b^	0.00641 ± 0.0003^c,d^	0.13
	C-Mg (8)	0.00135 ± 0.0002	0.00145 ± 0.0003	0.44
	S-Mg (8)	0.00218 ± 0.0005	0.00199 ± 0.0007	0.57
KW		19.720	23.039	
d.f.		3	3	
*P*		< 0.001	< 0.0001	

Abbreviations: d.f., degrees of freedom; EB, Evans blue; KW, Kruskal–Wallis test statistic; *P*, approximate χ^2 ^*P *value. Groups: sham control (C), sham control MgSO_4_-treated (C-Mg), septic (S) and septic MgSO_4_-treated (S-Mg). Data are expressed as means ± standard deviation.

Dunn's multiple comparisons test: ^a^septic versus sham control, *P *< 0.01; ^b^septic versus sham control MgSO_4_-treated, *P *< 0.001; ^c^septic versus sham control, *P *< 0.001; ^d^septic versus sham control MgSO_4_-treated, *P *< 0.001. A Mann–Whitney test was used for within-group comparisons. Two-tailed *P *values are shown in the last column.

**Table 3:** Assessment of edema by specific gravity of brain tissue

Measurement	Group (*n*)	Left hemisphere	Right hemisphere	*P*
SG	C (8)	1.0444 ± 0.0001	1.0443 ± 0.0002	0.24
	S (8)	1.0429 ± 0.0009^a,b^	1.0424 ± 0.0012^c,d^	0.44
	C-Mg (8)	1.0444 ± 0.0002	1.0444 ± 0.0001	0.44
	S-Mg (8)	1.0438 ± 0.0007	1.0439 ± 0.0004^e^	0.24
KW		18.831	24.724	
d.f.		3	3	
*P*		< 0.001	<0.0001	

Abbreviations: d.f., degrees of freedom; KW, Kruskal–Wallis test statistic; *P*, approximate χ^2 ^*P *value; SG, specific gravity. Groups: sham control (C), sham control MgSO_4_-treated (C-Mg), septic (S) and septic MgSO_4_-treated (S-Mg). Data are expressed as means ± standard deviation.

Dunn's multiple comparisons test: ^a^septic versus sham control, *P *< 0.001; ^b^septic versus sham control MgSO_4_-treated, *P *< 0.01; ^c^septic versus sham control, *P *< 0.01; ^d^septic versus sham control MgSO_4_-treated, *P *< 0.001; ^e^septic MgSO_4_-treated versus sham control MgSO_4_-treated, *P *< 0.05. A Mann–Whitney test was used for within-group comparisons. Two-tailed *P *values are shown in the last column.
